# Human Intestinal Parasitic Infections: Prevalence and Associated Risk Factors among Elementary School Children in Merawi Town, Northwest Ethiopia

**DOI:** 10.1155/2021/8894089

**Published:** 2021-02-15

**Authors:** Destaw Damtie, Baye Sitotaw, Sissay Menkir, Bizuayehu Kerisew, Kedir Hussien

**Affiliations:** Bahir Dar University, College of Science, Department of Biology, Bahir Dar, Ethiopia

## Abstract

**Background:**

Intestinal parasitic infection is still common in Ethiopia. Periodic evaluation of the current status of human intestinal parasitic infections (HIPIs) is a prerequisite to controlling these health threats. This study is aimed at assessing the prevalence and determinant factors of HIPIs among elementary school-age children in Merawi town.

**Methods:**

A school-based cross-sectional study design was used among 403 children. The direct wet mount method was used to diagnose the stool samples. The sociodemographic and behavioral characteristics of the respondents were collected using structured questionnaires. The data were analyzed using the chi-square test and logistic regression.

**Results:**

Out of the 403 students, the overall prevalence of HIPIs was 173 (42.9%). The magnitudes of single and double infections were 39.7% and 3.2%, respectively. Seventy-two (17.9%) were positive for *Entamoeba histolytica*, 63 (15.4%) for *Giardia lamblia*, 28 (9.6%) for *Ascaris lumbricoides*, 22 (6.9%) for hookworm, and 1 (0.2%) for *Schistosoma mansoni*. The prevalence of intestinal parasites was high in the age group of 6–11 years compared to other age groups. The following were the risk factors associated with HIPIs: groups aging 6 to 11 (AOR = 9.581; 95% CI: 0.531-17.498; *P* = 0.008), aging 12 to 18 (AOR = 3.047; 95% CI: 0.055-1.828; *P* = 0.008), not washing of hands after defecation (AOR = 3.683; 95% CI; 1.577-8.598; *P* = 0.003), not regularly washing of hands after defecation (AOR = 2.417; 95% CI; 1.224-4.774; *P* = 0.003), dirty fingernails (AOR = 2.639; 95% CI: 1.388-5.020; *P* = 0.003), not wearing shoes (AOR = 2.779; 95% CI: 1.267-6.096; *P* = 0.011), rural residence (AOR = 6.6; 95% CI; 0.06-0.351; *P* < 0.0001), and a family size greater than or equal to five (AOR = 2.160; 95% CI: 1.179-3.956; *P* = 0.013).

**Conclusion:**

The prevalence of HIPIs among elementary school children in Merawi town was very high. Thus, there is a need for intensive health education for behavioral changes related to personal hygiene and mass treatment for effective control of HIPIs in the study area.

## 1. Introduction

Human intestinal parasitic infections (HIPIs) have been a worldwide public health threat [1]. Such infectious diseases are already identified as neglected tropical diseases (NTDs) [2–5] and received attention very recently. Total control of the transmission of HIPIs and the reduction of possible aggravating factors are among the components of the sustainable development goals of the United Nations (2030 Agenda; Goal 3.3). Despite the efforts, intestinal parasites remain to be public health burdens, specifically in the tropical and subtropical regions [2, 3, 5].

Several risk factors for HIPIs in poor communities have been well documented [6]. Among others, poverty-related factors (poor sanitation, scarcity of potable water, unsafe human waste disposal systems, and open-field defecation), conducive environmental conditions for the parasites, lack of adequate health services, and low level of awareness are the contributing factors for the high rate of HIPIs [7–9].

School and preschool children are highly venerable to HIPIs due to exposure to the parasites as the result of their behaviors. Thus, urgent treatment and preventive interventions are required [4]. Young children have a less developed immune system, poor personal hygiene, and the habit of playing on contaminated soil. HIPIs cause health problems such as the increased risk of protein energy malnutrition, iron deficiency anemia, growth retardation, faulty cognitive development, and predisposition to other infections in young children [4, 10, 11].

There is a high global burden of protozoan intestinal parasites. For instance, *E. histolytica* infected some 48 million individuals. In the same way, there was a high global prevalence of giardiasis [4]. Besides, parasitic worms such as roundworm, hookworm, and whipworm have been among the common parasites affecting communities in low-income countries [3–5, 12]. Thus, protozoan parasites and soil-transmitted helminths have been resulting in high morbidities and mortality of children in sub-Saharan African countries [4]. Previous reports show a high frequency of HIPIs in this African region, affecting nearly all people once or several times during their life spans.

The Federal Ministry of Health of Ethiopia has been trying to halt HIPIs and several other diseases by training thousands of health extension workers and assigning them to every village. Despite the efforts, Ethiopia is still under the high burden of HIPIs [8, 13]. In Ethiopia, high prevalence rates of HIPIs (as high as 84%) were reported among primary school children [13–17].

Based on information obtained from health offices and clinics, people of Merawi town visit health centers mainly as the result of HIPIs. However, there was no study conducted on the prevalence of HIPIs and associated risk factors in Merawi town. Therefore, this study is aimed at scientifically documenting the prevalence and associated risk factors of HIPIs among elementary school children in Merawi town.

## 2. Material and Methods

### 2.1. The Study Area and Period

This study was conducted from March to April 2017 among students from three selected primary schools in Merawi town, Mecha district, Amhara Regional State, Northwest Ethiopia. All the elementary schools had toilet and water facilities, although not proportional to the number of students. Mecha district lies on 156,027 hectares of land. It is located at an elevation of 1,800–2,500 m above sea level.

Mecha district is bordered with Yilmana Denisa, Achefer, Sekela, and Bahir Dar administration districts on the East, West, South, and North, respectively. The mean annual rainfall and temperature range of Mecha district are 1,500 to 2,200 mm and 24 to 27°C, respectively. This district has two climatic conditions: “Weinadega” (80%) and “Dega” (20%). Its geographical structure is plain (75%), mountain (13%), and valley (4%) (Mecha District Information and Communication Center Department, 2011/12).

The study area lies at 523 km Northwest of Addis Ababa (the capital of Ethiopia) and 35 km southwest to Bahir Dar (the capital of the Amhara Regional State). According to the 2007 population census, the total population of the district was 292,250 (147,700 males, 144,550 females) [18]. Close to a quarter (21%) (63,627) (30,606 males and 33,021 females) of the district's population is from Merawi town. Mecha district has 13 health centers, 46 health posts, and one hospital and 103 primary schools, seven secondary schools, and one preparatory school. Merawi town has five health facilities (one health center, three health posts, and one hospital) and five schools (three elementary, one secondary, and one preparatory). The majority of the population of the district is engaged in mixed agricultural activities. The residents earn their living as farmers, merchants, daily laborers, and government employees. The dominant crops grown are maize, millet, and, “teff”, and the animals reared were cattle, goats, sheep, and poultry. The inhabitants of the district use water sources from streams, rivers, wells or pools, and tap water.

### 2.2. Participants

All students enrolled in the three elementary schools (Merawi 01 Kebele, Merawi Junior, and Merawi 02 Kebele) in the 2016/2017 academic year used as sources of data for this study. The Merawi 01 Kebele Primary School had 3,712 students, Merawi Junior Primary School 1,929 students, and Merawi 02 Kebele Primary School 978 students. The study subjects were 403 participants of all age groups and both sexes.

### 2.3. Variables

#### 2.3.1. The Dependent Variable

Prevalence of human intestinal parasitic infections is the dependent variable.

#### 2.3.2. Independent Variables

Gender, age, the habit of handwashing before and after meals, the habit of handwashing after defecation, fingernail cleanliness, shoe wearing habit, residence, latrine facility, latrine usage, source of drinking water, treatment of water before drinking, family size per household, parental education, and parental occupation are the independent variables.

### 2.4. Inclusion and Exclusion Criteria

#### 2.4.1. Inclusion Criteria

Those volunteers who/whose guardians signed informed consent and delivered stool specimens and those who did not receive any antiparasitic treatments in the days before sampling are included.

#### 2.4.2. Exclusion Criteria

Students who did not have signed informed consent, those who did not properly collect their stool sample, students who did not answer the questions on the form for sample collection, and subjects who had taken antiparasitic drugs in the last three weeks or during data collection were excluded.

### 2.5. The Study Design and Sampling Technique

A cross-sectional study was conducted among students of three primary schools (Merawi 01 Kebele, Merawi Junior, and Merawi 02 Kebele) in Merawi town in 2017. Out of the 112 sections comprising of 6,619 students (3,311 males and 3,308 females), 14 sections were selected using the lottery system. Students' names from the attendances of the 14 sections were ordered alphabetically, from which the participants were selected by a simple random sampling technique.

### 2.6. Sample Size Determination

The sample size was estimated using the following statistical formula [18]:

(1) $$\begin{array}{c} n=\frac{Z^{2}}{d^{2}}p\left(1-p\right). \end{array}$$

where *n* = the minimum required sample size, *z* = 1.96 at 95% confidence interval, *p* = prevalence of intestinal parasites, and *d* = margin of the sampling error assumed to be 0.05. Since the overall prevalence rate (*p*) of intestinal parasites was not known in the study area, it was taken as 50% and this gave a minimum sample size of 384.

(2) $$\begin{array}{c} n=\frac{{\left(1.96\right)}^{2}}{{\left(0.05\right)}^{2}}\left(0.5\right)\;\left(1-0.5\right)=384. \end{array}$$

To lessen errors arising from the likelihood of noncompliance or possible dropout, 5% of the sample size (19 children) was added to the normal sample size. Consequently, 403 total school children were selected. Age-wise, the respondents were grouped as middle childhood (6 to 11 years), early adolescence (12 to 18 years), and late adolescence (19 to 21 years) [19].

### 2.7. Questionnaire Data Collection

Pretested standardized questionnaires were developed based on known potential risk factors. These questionnaires were constructed in English and translated into Amharic. Then, children were interviewed in their mother tongue. For those students who were not able to respond to questionnaires properly, their parents/guardians were contacted through school principals and gave interviews on behalf of their children. During the interview, the fingernails, the general hygienic conditions, and the footwear of students were inspected by the interviewers. Coded questionnaires were used to gather information on the demographic and socioeconomic characteristics of the participants.

### 2.8. Stool Sample Collection and Examination

The subjects were instructed to provide stool samples (10–20 g) and were provided labeled clean cartons, toilet tissue papers, and pieces of applicator sticks. The stool samples were labeled and microscopically examined using the direct wet mount method for protozoa, helminths, and intestinal parasites.

A direct wet mount was conducted as follows. A drop of normal saline (0.85% NaCl solution) was added at the center of a labeled slide. A small amount of fecal specimen was added in the saline solution and thoroughly emulsified using an applicator stick. After homogeneous thin films were prepared on each slide, coverslips were placed on each preparation and examined for parasites under a light microscope of 10× and 40× objectives.

### 2.9. Data Analysis

After the row data were collected through interviews and parasitological examinations, results were initially fed into Microsoft Excel 2007 software and then copied to Statistical Package for Social Sciences (SPSS) software version 20. Descriptive logistic regression was used to quantify the degree of association of HIPIs with socioeconomic and potential risk factors. The levels of significant differences of proportions were compared using the Pearson chi-square test. Logistic regression analysis was expressed as an odds ratio to evaluate the prevalence and associated with HIPIs. The 95% CI was used to show the accuracy of data analysis, and probabilities less than 5% (*P* < 0.05) were considered statistically significant.

## 3. Results

### 3.1. General Characteristics of the Study Participants

**Figure 1:**
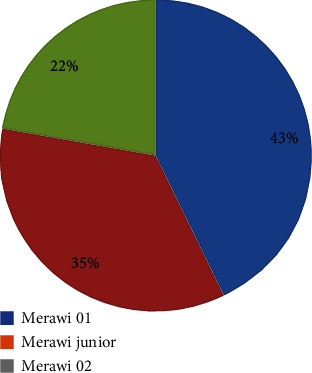
Proportion of participants from the three schools.

The demographic and socioeconomic characteristics of the participants are shown in Table 1. A total of 403 participants (49.2% male and 50.6% female) aged between seven and 20 years with a mean age of 11.9 were involved in this study. One hundred seventy-three (42.9%) were from Merawi 01 Kebele Primary School, 143 (35.5%) from Merawi Junior School, and 87 (21.6%) from Merawi 02 Kebele Primary School. The distribution of participants in the three schools is displayed in Figure 1. Two hundred sixty-five (65.8%) of the respondents regularly washed their hands before and after meals, 175 (43.4%) of them did not wash their hands after defecation, 258 (64.0%) of the students had clean fingernails, and 335 (83.1%) of the participants had shoe wearing habits. The majority of the respondents, 327 (81.1%), used to drink tap water, 372 (92.3%) of them used untreated water, 212 (52.6%) came from rural areas, 392 (97.3%) had toilet facilities, 278 (69.0%) regularly used latrines, 221 (54.8%) were from family sizes less than five (<5), 150 (37.2%) of the fathers had an education level of secondary school and above, and 180 (44.7%) of the mothers were illiterate. The majority of the fathers 196 (48.6%) were farmers and the majority of their mothers 193 (47.9%) were housewives.

### 3.2. Prevalence of HIPIs among the Study Participants

The overall prevalence of HIPIs was 173 (42.9%). The magnitudes of single and double infections were 160 (39.7%) and 13 (3.2%), respectively. The top three predominant intestinal parasites were *E*. *histolytica* (19.1%), *G. lamblia* (14.7%), and *A. lumbricoides* (6.4%) (Table 2). Ninety-four (54.3%) of the males, 79 (45.7%) of the females, 119 (60.4%) of those aging 6 to 11 years, 90 (65.2%) of those who washed their hands irregularly before and after meals, 110 (62.9) of the students who did not wash their hands after defecation, 93 (64.1%) of children with unclean fingernails, 42 (61.8%) of the students who did not have shoe wearing habits, 126 (59.4%) of the urban dwellers, 6 (54.5%) of the students who had no latrine facilities, 75 (60.0%) of those who sometimes use latrines, 7 (58.3%) of those who used river water, 33 (51.6%) of well water users, 161 (43.3%) of the untreated water users, 108 (59.3%) of the students from family sizes of greater than or equal to five, 37 (46.8%) of those with illiterate fathers, 110 (61.1%) of the students with illiterate mothers, 94 (48.0%) of those with farmer fathers, and 95 (49.2%) of the students with housewife mothers were positive for HIPIs (Table 1).

### 3.3. Factors Associated with Intestinal Parasite Positivity

The *X*^2^ analysis showed that, among the factors studied, variation in age, the habit of handwashing before and after meals, habit of handwashing after defecation, fingernail cleanliness, shoe wearing habits, residence, latrine usage, family size per household, and maternal education status had statistically significant associations with HIPIs (*P* < 0.05). The age group 6 to 11 years (*X*^2^ = 42.557; *P* < 0.0001), group not regularly washing hands before and after meals (*X*^2^ = 48.869; *P* < 0.0001), group not washing hands after defecation (*X*^2^ = 60.918; *P* < 0.0001), group dirty fingernails (*X*^2^ = 41.588; *P* < 0.0001), group not wearing of shoes (*X*^2^ = 11.847; *P* = 0.001), group living in rural areas (*X*^2^ = 42.829; *P* < 0.0001), group irregular use of latrines (*X*^2^ = 21.556; *P* < 0.0001), group family sizes of five or more (*X*^2^ = 36.490; *P* < 0.0001), and group having illiterate mothers (*X*^2^ = 45.598; *P* < 0.0001) were statistically associated with HIPIs (*P* < 0.05) (Table 1).

### 3.4. Risk Factors Associated with HIPIs

The strength of the association of HIPIs with their risk factors is presented in Table 3. The results from the univariate analysis showed that students in the age groups of 6 to 11 were five times (AOR = 4.455; 95% CI: 2.899-6846; *P* < 0.0001) more likely to be infected by HIPIs than students in the age group of 19 to 21. Students who irregularly washed their hands before and after meals were 4.111-fold (AOR = 4.111; 95% CI: 2.659-6.358; *P* < 0.0001) more likely to acquire HIPIs than those who regularly washed their hands.

Students who did not wash and who sometimes wash their hands after defecation were 8.839 times (AOR = 8.839; 95% CI: 4.759-16.419; *P* < 0.0001) and 3.195 times (AOR = 3.195; 95% CI: 1.994-5.119; *P* < 0.0001), respectively, more likely to be infected by HIPIs compared to those who regularly washed their hands after defecation. Students with unclean fingernails had 4.532 times HIPI risk (COR = 4.532; 95% CI: 2.865-7.167; *P* < 0.0001) than those with clean fingernails; students who did not wear shoes were 2.516 times more likely to be infected by HIPIs (COR = 2.516; 95% CI: 1.472-4.300; *P* = 0.001) than students who wore shoes regularly.

Rural dwellers were 4.021 times at higher risk of HIPIs than urban dwellers (COR = 4.021; 95% CI: 2.625-6.159; *P* < 0.0001). The HIPI rate among students who did not use latrines was 2.755 times higher (COR = 2.755; 95% CI: 1.785-4.253; *P* < 0.0001) than that with latrine users; the infection rate in students from a family size of more than or equal to five was 3.503 times (COR = 3.503; 95% CI: 2.316-5.298; *P* < 0.0001) higher than that in students from family sizes of less than five. Students with illiterate fathers and mothers were 1.309 (COR = 1.309; 95% CI: 0.766-2.237; *P* < 0.0001) and 4.532 (COR = 1.309; 95% CI: 2.865-7.167; *P* < 0.0001) times, respectively, more likely to be infected by HIPs than students with fathers and mothers above secondary education.

In the multivariate analysis, significant differences (*P* < 0.05) were observed with differences in age, the habit of handwashing after defecation, fingernail cleanliness, shoe wearing habits, residence, and family size per household. Students in the age groups of 6–11 and 12–18 were ten times (AOR = 9.581; 95% CI: 0.531-17.498; *P* = 0.008) and three times (AOR = 3.047; 95% CI: 0.055-1.828; *P* = 0.008), respectively, more likely to be infected by HIPIs than students in the age group 19–21. Students who did not wash and irregularly wash their hands after defecation were fourfold (AOR = 3.683; 95% CI; 1.577-8.598; *P* = 0.003) and twofold (AOR = 2.417; 95% CI; 1.224-4.774; *P* = 0.003), respectively, at a higher risk of HIPIs than students who regularly washed their hands.

Children who had dirty fingernails were three times (AOR = 2.639; 95% CI: 1.388-5.020; *P* = 0.003) at risk of HIPIs than children who had clean fingernails. Students who had no shoe wearing habit were close to three times more likely to be infected by HIPIs (AOR = 2.779; 95% CI: 1.267-6.096; *P* = 0.011) than students who wore shoes regularly. Students who were living in rural areas were seven times (AOR = 6.6; 95% CI; 0.06-0.351; *P* < 0.0001) more likely to be infected by HIPIs than those who used to live in the urban areas. Students who lived in family sizes greater than or equal to five were two times more likely to be infected (AOR = 2.160; 95% CI: 1.179-3.956; *P* = 0.013) than students who lived in family sizes of less than five.

## 4. Discussion

The overall prevalence of HIPIs among students from Merawi elementary schools was 42.9%. It was in line with the findings from Aksum town (Northern Ethiopia) (44.6%) [20], Gurage Zone (South Ethiopia) (42.1%) [21], and a study done in Amalapuram (Nepal) (49%) [20]. However, it was lower than the findings from Dagi Primary School (Amhara National Regional State) (77.9%) [21] and Delgi (Northwest Ethiopia) (79%) [22]. It was higher than that of the studies in Gondar (Northwest Ethiopia) (34.2%) [23] and Arba Minch (Southern Ethiopia) (27.7%) [24]. The difference in prevalence could be due to differences in population density, sanitary facilities and practices, public health practices, sanitation of food and water, malnutrition, host resistance, availability of safe drinking water, socioeconomic differences, and environmental changes.

According to this finding, males were more infected than females. This finding is similar to that of the studies conducted in Chilga (Northwest) [25] and Delgi [22]. In contrast, in a study from Adigrat, females were more infected than males [26].

A higher prevalence of HIPI has been recorded in Merawi 01 Kebele with 98 (24.3%) compared to 65 (16.1%) and 23 (5.7%) in Merawi Junior and Merawi 02 Kebele Primary School, respectively. The trend of HIPIs reduced with an increase in age. For instance, from 60.4% in the age group 6 to 11 years to 25.5% in the age group 12 to 18 years. The reason might be due to older children had better awareness of washing hands and personal hygiene compared to the younger ones [23].

Among the intestinal parasites identified, *E. histolytica* (19.1%) was the dominant one. This result was in agreement with the findings in Aksum (Tigray) (17%) [24], Motta (Western Amhara) (17%) [27], and Sohag Governorate (Egypt) (20.4%) [28]. This prevalence was higher than the reports from Debre Elias (6.7%) [29] and Delo Mena (5%) districts [30]. However, it was lower than that of the studies conducted in Dona Berber (Bahir Dar town) (24.5%) [15] and Delgi (North Gondar) (27%) [26]. The possible reasons for these differences might be associated with the differences in handwashing practices, close contact with domestic animals [31], the sanitary status of foods and waters, the sanitation of food handlers, and the status of malnourishment.

The second most prevalent intestinal parasite identified in this study was *G. lamblia* (17.7%). This finding was close to the reports from Aksum town (Tigray) (14%) [24], Dona Berber (Bahir Dar) (11.4%) [15], and Sohag Governorate (Egypt) (15.2%) [28]. It was higher than the reports from Adigrat town (2.28%) [26], around Langano (6.2%) [32], and Nepal (5.76%) [33]. However, it was much lower than the studies in Delgi (North Gondar) (41.9%) [22], Gurage Zone (South Ethiopia) (47.7%) [34], and Dagi Primary School (Amhara National Regional State) (23.6%) [21]. Differences in the timings of the studies and geographical locations may be justifications for these variations.

The third most prevalent intestinal parasite identified by the current study was *A. lumbricoides* (6.4%). This prevalence was in line with the works in Gondar (Northwest Ethiopia) (6.5%) [23], Aksum town (9%) [24], and Sohag Governorate (Egypt) (6.5%) [28]. However, it was greater than the studies in Debre Elias (0.6%) [29] and in Nepal (2.3%) [33] and was much lower than the studies in Tilili (39.7%) [35], Delgi (48%) (North Gondar) [22], and Lumame (32.6%) [36]. The possible reasons for *A. lumbricoides* prevalence might be due to differences in awareness about the modes of transmission of the parasite, personal and environmental hygiene, and handwashing practice after the toilet.

The prevalence of hookworm in the present study (5.7%) was higher than that of the studies conducted by Abera [35] and Nibret in Tilili (0.5%) and Haftu *et al*. [37] in Arba Minch (2.2%). This difference might be due to the differences in awareness of wearing shoes and a large family size. The prevalence of hookworm was much lower than that from the reports from Dagi Primary School, Amhara National Regional State (23.6%) [21], in Dona Berber, Bahir Dar (22%) [15], and in Zegie Peninsula (43.4%) [14]. This difference might be due to the geographical location of the study areas. However, hookworm prevalence was comparable with that of the work of EL-Masry *et al*. [28] in Sohag Governorate (Egypt) (5.1%).

The prevalence of *S. mansoni* (0.2%) in the present study was very much lower than that in Delgi (North Gondar) (15.9%) [22], in Mek'ele city [38] (23.9%), and Zegie Peninsula [14] (29.9%). This difference might be due to differences in the residences of the study subjects and living and socioeconomic conditions.

The results in the present study showed that subjects had single and double infections. The mixed infections detected were *E. histolytica* and *G. lamblia* (1.48%), *E. histolytica* and hookworm (0.98%), *A. lumbricoides* and hookworm (0.49%), and *E. histolytica* and *A. lumbricoides* (0.25%). This result agrees with the works from Aksum town [24] and Dona Berber (Bahir Dar) [15].

A strong association existed between HIPIs and the residence of students, fingernail cleaning, handwashing habit after defecation, shoe wearing habit, and family size (*P* < 0.05). This study was in agreement with the studies done in Dona Berber (Bahir Dar) [15] and Al-Ahsa (Saudi Arabia) [39].

Students from rural areas were almost seven times (AOR = 6.666; 95% CI: 0.06-0.351; *P* < 0.0001) more likely to get HIPIs compared with students from Merawi (town). This finding agreed with the findings of the studies conducted in Motta (Amhara Region) [27] and Adigrat [26]. However, it was different from the work in Delgi [22]. The possible reasons for more infections among students from rural areas might be the presence of relatively poor personal and environmental hygiene, poor handwashing habits, presence of unclean fingernails, and a large family size.

The risk of HIPIs among students who did not frequently wash their hands after defection was almost four times (AOR = 3.683; 95% CI: 1.577-8.598; *P* = 0.004) more than that of those who used to wash their hands regularly. Students who sometimes washed their hands after defecation were two times (AOR = 2.417; 95% CI: 1.224-4.774; *P* = 0.004) more at risk than students who used to wash their hands regularly. This was comparable with the studies conducted in Motta (Amhara Region) [27] and Dona Berber (Bahir Dar) [15].

Likewise, the likelihood of being infected by intestinal parasites among students who did not frequently wear shoes was three times more than that among students who regularly used to wear shoes (AOR = 2.776; 95% CI: 1.312-5.873; *P* = 0.008). Similar associations between HIPIs and shoe wearing habits of students were reported from Dagi Primary School, Amhara National Regional State [21], Dona Berber (Bahir Dar) [15]. The possible explanation may be that walking barefoot increases the chance of being infected with IPs, especially those which enter the body through skin penetration.

At the same time, the risks of being infected by IPs were increased almost three times (AOR = 2.639; 95% CI: 1.388-5.020; *P* = 0.003) more among students with unclean fingernails as compared to students who had clean fingernails. Similar associations of HIPIs with unclean fingernails were reported from Lumane [36] and Dona Berber (Bahir Dar) [15].

Furthermore, the family size was strongly associated with HIPIs. The likelihood of being infected by HIPs was increased by two times (AOR = 2.160; 95% CI: 1.179-3.956; *P* = 0.013) more among students belonging to the family size of five and above as compared to students in a family size of less than five. This agrees with studies conducted in Tilili [35] and Dona Berber (Bahir Dar) [15].

## 5. Conclusions

A relatively high prevalence of HIPIs (42.9%) was observed among students from primary schools in Merawi town. Single infections accounted for 39.7% of the infections followed by double infections (3.2%). HIPI was common among children of middle childhood (6 to 11 years) than early adolescent (12 to 18 years) and late adolescent (19 to 21 years) students. Male children were more prone to HIPI than female children. The age 6 to 11 years, poor handwashing habit after defecation, dirty fingernails, lack of shoe wearing habit, rural residence, and family size greater than or equal to five were risk factors associated with HIPIs.

## 6. Recommendation

Owing to the high prevalence of HIPIs in the present study, awareness creation to the children and their guardians, establishment of sanitary facilities in the school compounds, inspecting the sanitary situations of students especially in the age group of 6 to 11 years, monitoring the hand washing and finger trimming habits of students, and promoting shoe wearing and sanitary measures in the rural areas are recommended.

## 7. Limitations

Only the wet mount technique was used to identify the parasites. This may underestimate the prevalence of HIPs in the study area compared to the more efficient and specific techniques such as formal ether concentration and Kato Katz methods.

## Figures and Tables

**Table 1:** Informant characteristics of elementary school children in Merawi town and their associations with HIPIs (April 2017).

Risk factors	No. (%)	HIPIs		*X* ^2^	*P* value
		Positive no. (%)	Negative no. (%)		
Gender					
Male	199 (49.2)	94 (54.3)	105 (45.7)	2.978	0.084
Female	204 (50.6)	79 (45.7)	125 (54.3)		
Total	403 (100)	173 (42.9)	230 (57.1)		
Age					
6–11	197 (48.9)	119 (60.4)	78 (39.6)	48.869	*P* < 0.0001^∗^
12–18	196 (48.6)	50 (25.5)	146 (74.5)		
19–21	10 (2.5)	4 (40)	6 (60)		
Total	403 (100)	173 (42.9)	230 (57.1)		
The habit of handwashing before and after meals					
Always	265 (65.8)	83 (31.3)	182 (68.7)		
Sometimes	138 (34.2)	90 (65.2)	48 (34.8)	42.557	*P* < 0.0001^∗^
Total	403 (100)	173 (42.9)	230 (57.1)		
The habit of handwashing after defecation					
Always	95 (23.6)	16 (16.8)	79 (83.2)		
Sometimes	133 (33.0)	47 (35.3)	86 (64.7)		
Not at all	175 (43.4)	110 (62.9)	65 (37.1)	60.918	*P* < 0.0001^∗^
Total	403 (100)	173 (42.9)	230 (57.1)		
Fingernail cleanliness					
Clean	258 (64.0)	80 (31.0)	178 (69.0)		
Not clean	145 (36.0)	93 (64.1)	52 (35.9)	41.588	*P* < 0.0001^∗^
Total	403 (100)	173 (42.9)	230 (57.1)		
Shoe wearing habit					
Yes	335 (83.1.)	131 (39.1)	204 (60.8)		
No	68 (16.9)	42 (61.8)	26 (38.2)	11.847	0.001^∗^
Total	403 (100)	173 (42.9)	230 (57.1)		
Residence					
Rural	212 (52.6)	126 (59.4)	86 (40.6)	42.829	*P* < 0.0001^∗^
Urban	191 (47.4)	47 (24.6)	144 (75.4)		
Total	403 (100)	173 (42.9)	230 (57.1)		
Latrine facility					
Present	392 (97.3)	167 (42.6)	225 (57.4)	0.623	0.430
Absent	11 (2.7)	6 (54.5)	5 (45.5)		
Total	403 (100)	173 (42.9)	230 (57.1)		
Latrine usage					
Always	278 (69.0)	98 (35.3)	180 (64.7)		
Sometimes	125 (31.0)	75 (60.0)	50 (40.0)	21.556	*P* < 0.0001^∗^
Total	403 (100)	173 (42.9)	230 (57.1)		
Source of drinking water					
Tap water	327 (81.1)	133 (40.7)	194 (59.3)	4.110	0.128
River/stream	12 (3)	7 (58.3)	5 (41.7)		
Well water	64 (15.9)	33 (51.6)	31 (48.4)		
Total	403 (100)	173 (42.9)	230 (57.1)		
Treatment of water before drinking					
Boiled	4 (1.0)	3 (75)	1 (25)	3.587	0.310
Filtered	21 (5.2)	6 (28.6)	15 (71.4)		
Chemical treated	6 (1.5)	3 (50)	3 (50)		
Untreated	372 (92.3)	161 (43.3)	211 (56.7)		
Total	403 (100)	173 (42.9)	230 (57.1)		
Family size per household					
<5	221 (54.8)	65 (29.4)	156 (70.6)		
≥5	182 (45.2)	108 (59.3)	74 (40.7)	36.490	*P* < 0.0001^∗^
Total	403 (100)	173 (42.9)	230 (57.1)		
Paternal education					
Illiterate	79 (19.6)	37 (46.8)	42 (53.2)	1.080	0.058
Primary	174 (43.2)	70 (40.2)	104 (59.8)		
≥secondary	150 (37.2)	66 (44.0)	84 (56.0)		
Total	403 (100)	173 (42.9)	230 (57.1)		
Maternal education					
Illiterate	180 (44.7)	110 (61.1)	70 (38.9)	45.598	*P* < 0.0001^∗^
Primary	167 (41.4)	43 (25.7)	124 (74.3)		
≥secondary	56 (13.9)	20 (35.7)	36 (64.3)		
Total	403 (100)	110 (61.1)	70 (38.9)		
Paternal occupation					
Farmer	196 (48.6)	94 (48.0)	102 (52.0)	6.314	0.120
Merchant	25 (6.2)	12 (48.0)	13 (52.0)		
Government employed	43 (10.7)	19 (44.2)	24 (55.8)		
Private	139 (34.5)	48 (34.5)	91 (65.5)		
Total	403 (100)	94 (48.0)	102 (52.0)		
Maternal occupation					
Farmer	51 (12.6)	20 (39.2)	31 (60.8)	7.421	0.115
Merchant	21 (5.2)	8 (38.1)	13 (61.9)		
Government employed	26 (6.5)	12 (46.2)	14 (53.8)		
Private	112 (27.8)	38 (33.9)	74 (66.1)		
Housewife	193 (47.9)	95 (49.2)	98 (50.8)		
Total	403 (100)	20 (39.2)	31 (60.8)		

^∗^Statistically significant at *P* < 0.05.

**Table 2:** The prevalence of HIPIs among elementary school children in Merawi town, from March to April 2017.

IPIs	No. of infected	Percent (%)
Single infection		
*E*. *histolytica*	66	16.4
*G. lamblia*	53	13.2
*A. lumbricoides*	23	5.7
Hookworm	17	4.2
*S*. *mansoni*	1	0.2
Double infection		
*E*. *histolytica* + *G. lamblia*	6	1.5
*E*. *histolytica +* hookworm	4	1.0
*A. lumbricoides +* hookworm	2	0.5
*E*. *histolytica + A. lumbricoides*	1	0.2

**Table 3:** Bivariate and multivariate analysis of potential risk factors associated with HIPIs among elementary school children in Merawi town.

Risk factors	Parasitic infection		Univariate logistic regression		Multivariate logistic regression	
	Positive no. (%)	Negative no. (%)	COR (95%)	*P* value	AOR (95%)	*P* value
Age						
6–11	119 (60.4)	78 (39.6)	4.455 (2.899-6846)	*P* < 0.0001^∗^	9.581 (0.531-17-498)	*P* = 0.008^∗^
12–18	50 (25.5)	146 (74.5)	2.288 (0.119-1.599)		3.047 (0.055-1.828)	
19–21	4 (40)	6 (60)	1		1	
Total	173 (42.9)	230 (57.1)				
The habit of handwashing before and after meals						
Always	83 (31.3)	182 (68.7)	1	*P* < 0.0001^∗^		
Sometimes	90 (65.2)	48 (34.8)	4.111 (2.659-6.358)			
Total	173 (42.9)	230 (57.1)				
The habit of handwashing after defecation						
Always	16 (16.8)	79 (83.2)	1	*P* < 0.0001^∗^	1	*P* = 0.003^∗^
Sometimes	47 (35.3)	86 (64.7)	3.195 (1.994-5.119)		2.417 (1.224-4.774)	
Not at all	110 (62.9)	65 (37.1)	8.839 (4.759-16.419)		3.683 (1.577-8.598)	
Total	173 (42.9)	230 (57.1)				
Fingernail cleanliness						
Clean	80 (31.0)	178 (69.0)	1	*P* < 0.0001^∗^	1	*P* = 0.003^∗^
Not clean	93 (64.1)	52 (35.9)	3.97 (2.589-6.116)		2.639 (1.388-5.020)	
Total	173 (42.9)	230 (57.1)				
Shoe wearing habit						
Yes	131 (39.1)	204 (60.8)	1	*P* = 0.001	1	*P* = 0.011^∗^
No	42 (61.8)	26 (38.2)	2.516 (1.472-4.30)		2.779 (1.267-6.096)	
Total	173 (42.9)	230 (57.1)				
Residence						
Rural	126 (59.4)	86 (40.6)	4.02 (2.625-6.159)	*P* < 0.0001	6.6 (0.06-0.351)	*P* < 0.0001^∗^
Urban	47 (24.6)	144 (75.4)	1		1	
Total	173 (42.9)	230 (57.1)				
Latrine usage						
Always	98 (35.3)	180 (64.7)	1			
Sometimes	75 (60.0)	50 (40.0)	2.755 (1.785-4.253)	*P* < 0.0001^∗^		
Total	173 (42.9)	230 (57.1)				
Family size per household						
<5	65 (29.4)	156 (70.6)	1	*P* < 0.0001^∗^	1	*P* = 0.013^∗^
≥5	108 (59.3)	74 (40.7)	3.503 (2.316-5.298)		2.160 (1.179-3.956)	
Total	173 (42.9)	230 (57.1)				
Paternal education						
Illiterate	37 (46.8)	42 (53.2)	1.309 (0.766-2.237)	*P* < 0.0001^∗^		
Primary	70 (40.2)	104 (59.8)	1.121 (0.649-1.938)			
≥secondary	66 (44.0)	84 (56.0)	1			
Total	173 (42.9)	230 (57.1)				
Maternal education						
Illiterate	110 (61.1)	70 (38.9)	4.532 (2.865-7.167)	*P* < 0.0001^∗^		
Primary	43 (25.7)	124 (74.3)	2.829 (1.517-5.276)			
≥secondary	20 (35.7)	36 (64.3)	1			
Total	110 (61.1)	70 (38.9)				

^∗^Statistically significant *P* value < 0.05.

## Data Availability

All datasets generated and analyzed during the study are presented in the text.

## Abbreviations

AOR: Adjusted odds ratio
CI: Confidence interval
COR: Crude odds ratio
NTDs: Neglected tropical disease
HIPIs: Intestinal parasitic infections
SPSS: Statistical Package for Social Sciences.
